# Indents

**Published:** 1908-02-15

**Authors:** 


### INDENTS.

☞Brief Articles of Techinical or Scientific Value will receive notice and comment. Contributions invited.

**Contouring Artificial Dentures.** The tone of the voice can be very greatly modified by the form of the denture which is fitted in the mouth. In one case recently I found that the contour of the denture back of the anterior teeth was not correct. I removed the denture, built on wax at the point indicated, removed some opposite the cuspid teeth, and by a little manipulation was able to correct the defect. The practical side of this subject consists in constructing dentures that will not by reason of their form or contour interfere with normal sound production.—J. H. PROTHERO, in *Review*.

**Unsolved Problems in Bridge-Work.** First, should the pulps in teeth which are to support artificial crowns be devitalized? Second, should a crown be made with a band, or without? Third, should a band, when one is used, extend beneath the gum margin, or not? Fourth, should we destroy or mutilate the crown of a sound tooth for the purpose of obtaining support for a bridge, or not? Fifth, if this is not warrantable, should we use an open-face crown, a so-called "hood" or "groove" attachment, or any other method? Sixth, should we use a "fixed" or a "removable" structure in the building of our bridges? —Dr. Goslee.

**Failure in Tooth Extraction.** Edwin N. Kent, D.M.D., of Brookline, Mass., suggests that a prominent cause of failure in the operation of extraction, particularly the extraction of roots which present but little substance above the alveoli, is "dull forceps." The point of the beaks on extracting forceps should be as carefully and almost as frequently sharpened as cutting instruments. Attention to this detail will save the patient much pain and the operator much embarrassment, as a forcep with a dull, rounded beak will not take hold firmly unless the grip is taken further rootward than is necessary with a well-sharpened instrument.—*Dental Brief*.

**Poison in Cigarettes.** Amerson maintains that the poison in cigarettes is manufactured in the cigarette as it is smoked. He says that in the smoking of the cigarette carbon monoxid is formed, and the inhalation of this gas is what does the harm. The smoke from cigars and pipes is so strong that it cannot be inhaled. This is not true of the cigarette. Amerson says that he has never known of heard of a cigarette smoker being hurt who did not inhale the smoke. He thinks that the evidence is conclusive that the poison in cigarette smoking is due to the smothered fire forming carbon monoxid, and this being inhaled, little by little, kills the smoker.—*Jour. Amer. Assn.*

**Partial Impressions.** The taking of partial impressions is simplified by making a core for the places where teeth have tipped together. To do this, I use a piece of sheet lead cut to the right size for a tray, and with a small amount of plaster or hard modelling compound take an impression of the undercut, removing lead, trimming core to right size and shape, slightly oiling it and the adjoining teeth and proceed as for a full case, except I oil the tray so it can be removed and the buccal part of the impression broken in as few pieces as possible; the lingual coming away in one piece. The core is then taken out and, with the pieces, adjusted in the tray and waxed together.—C. L. SMITH, in *Review*.

**Instruction in Oral Sanitation.** A conservative estimate would place the number of dentists who give any instructions in oral sanitation or who require their patients to observe any form of prophylectic *regime*, at less than ten per cent. The average dental practitioner does not know what a clean mouth should look like. It is not uncommon to hear a patient say that his dentist told him that the stains on his teeth were a protection, and should not be removed. It is still more common to hear him say that he has been warned against the too frequent use of abrasive powders, for fear that he might wear the enamel off his teeth. Many laymen give similar excuses for not brushing the teeth regularly.—Dr. Corley.

**Use of the Blue Light.** The contrivance is a simple 16-candle power blue electric light globe arranged in a funnel-shaped tin shield which, at its mouth, is about four inches in diameter; this is extended about four inches and has at its end a round blue glass and convex lens. The round blue glass is used to disseminate the blue rays so that the patient may not know the simplicity of the apparatus, and I attribute no especial virtue to the lens. Dr. Watkins claims that in cases of acute abscess, impacted third molars and their associate lesions he has used the blue light with great effect, relieving the pain and causing the disappearance of the inflammatory condition usually attending this form of disease.—*Items of Interest.*

**To Insert an Amalgam Filling as an Inlay.** The cavity should be prepared and made dry with rubber dam, etc. The cavity is then smeared with a rather thin cement mix. A lining of gold foil is then adapted to the cavity by burnishing or otherwise, expelling all but enough of the cement to attach the gold to the cavity. The margins of the gold foil are then trimmed flush, and the amalgam pocket into the gold lined cavity. The amalgam unites with the gold inlay matrix which the cement has attached to the tooth. By burnishing the margin carefully no cement or gold will be evident. The mercury in the amalgam will not attack the dentine or enamel, and we get a much better filling than if amalgam alone were used.—Dr. Vance, in *Tri-State Journal.*

**Relative Solubilities of Cements.** Dr. J. O. Keller, in the February *Dental Review*, says: Cements calcined in the manufacture at no higher temperature than 2,000 degrees will be dissolved from the teeth much more readily than cements which have been subjected to a heat of 2,800 degrees in calcination. Acids dissolve the low calcined cements readily because their particles are smaller and are not densely condensed by a vitrifying heat. These cements are more porous and crystaline in character. The alkilies of the mouth have a strong affinity for the soda phosphate, and consequently dissolve the porous and low fusion powders more readily. In the highly calcined cements the powders are not soft, but flint-like and do not yield readily to the action of the acid or alkiline solvents.

**Orthodontia: Adenoids.** Dr. Earnest W. Vickers, in a paper upon "Orthodontia," read before the Odontological Society of Victoria, Melbourne, Australia, charges the infant's comforter, where it has been used continuously, with being responsible for 95 per cent of adenoid conditions. He explains that the child's lips pass over the areolæ of the natural breast, and being pressed against the breast, all air is excluded from the mouth, and sucking is a normal, passive action. On the contrary, when the comforter is in use, an abnormal lip pressure is required during sucking, air passes in at the corners of the mouth, and as a result the act of sucking imposes greater effort on the tissues concerned. This, he contends, leads to hypertrophied tonsils and adenoid growths.—*Australian Journal of Dentistry*, May, 1907, page 161.

**Retaining Teeth by Means of Inlays.** In cases where the teeth have become loosened by the ravages of pyorrhea or by mechanical injuries, an excellent use can be made of gold inlays. Two or more teeth can be held firmly in place by an appliance made of two or more inlays soldered together and cemented in place. In cases where two teeth have become separated, allowing a space for food to lodge and cause an inflammatory and diseased condition of the tissues, which, as we all so well know, is the source of a great deal of trouble, can be corrected very satisfactorily by the use of two gold inlays, set mesio-distally and united or soldered at the occlusal angle, thereby retaining the two teeth in place, preventing the space from becoming larger, and closing it from any further wedging of food.—*E. M. S. Fernandez, Chicago.*

**Silicate Cements.** A writer, in whom I place greater confidence, says the Ascher powder is a silicate of aluminum, with a slight quantity of beryllium in it, and that the use of beryllium forms the basis of the Ascher patents. He states that the silicon, beryllium and aluminum oxid are fused at a very high temperature and the resultant mass ground to an inert, amorphous powder; that the cement resembles a hydraulic Portland cement with a binder of metal phosphates instead of hydrates; and that the cement may be sited as an orthophospho-silicate of aluminum, calcium and beryllium. Whether this be true or not the manufacturers alone can say, and so far they have not said. In their circular of information they state that the powder contains about fifty-four per cent of silicon and other "light and practically unknown metals," including beryllium.—G. E. Hunt, in *Dental Era.*

**Dilute Hydrochloric Acid in the Amalgamation of Dental Alloys.** I do not think you can properly amalgamate alloys unless you use an acid aqueous solution in the mixing. This point was given to me by the late Dr. Clapp, of Boston, some seven or eight years ago, and I have since employed the following method: Take hydrochloric acid, one part, and twenty parts of water; place the mercury in the mortar and pour on a sufficient quantity of alloy; then cover that with the acid solution, and you will be astonished to find how quickly the alloy will amalgamate. At the same time the dross is removed, and perfectly clean amalgam will be the result. After amalgamating it thoroughly, wash it off in the mortar with clear water, and then drying it on a napkin, you have a very clean alloy, and one that keeps bright in the mouth.—G. A. MAXFIELD, in *Dental Cosmos.*

**Gold and Amalgam in Combination.** In many instances of extreme decay in molars and bicuspids, gold foil will preserve such teeth when it is placed in apposition to amalgam on the dental surfaces, much better than gold will alone. When I see some of these fillings that were put in twenty-five and thirty years ago, standing up well to-day, I am convinced that there is a field for the combination of gold and amalgam in the preservation of teeth. I am not sure yet of the true explanation, but my theory is that by putting the two metals together we have a short circuiting of electrical current which almost always results in a beautiful oxidation of the baser metal and clean surface of gold, and as a result of this short circuiting there is an electrical impulse which is inimical to the growth of bacteria. That has been proven so conclusively that we can state it as a fact.—DR. C. P. PRUYN, in *Review.*

**To Cap a Badly Broken Molar Crown.** After cementing two strong pivots into the canals, cover them with gutta percha to allow of taking an impression in the usual way. After the tooth is built up by hard wax to its correct outlines, with perfect contact points and sharp occlusions, with a spoon excavator, cut a big hole in the center of the matrix and hollow out the wax. This can be done whenever desirable in all big gold inlays, thus making ho - low gold inlays. As the investment material will fill up this excavation, the result will be a perfect fitting gold crown, avo ding unnecessary cutting, preserving the bulky portion of the tooth, doing away with the free edges of a band irritating the gum, having on the contrary perfect smooth edges, allowing of easy control, the contact points more perfect than in case of a band and a strong biting surface that will never wear out.—O. SOLBRIG, in *Review*.

**Short-Cut Methods.** "Short-cut methods" usually mean short-cut results, and upon such too strong a condemnation cannot be pronounced. There is a common significance given to this term "short-cut methods," which means a saving of time and material, and which gives a fair to excellent external semblance; hence a perpetual fraud. In contradistinction to short-cut methods we may have modern scientific methods because the results are more durable, esthetic and conducive to health. No new methods should be adopted unless the service rendered the patient is equally as good or better than by the old one.

Barring the personal equation, we believe the three causes, "We practice prosthesis empirically," A wrong conception of the possibilities of artificial dentures," and "Cheap materials and short-cut methods," will account for all our failures in prosthesis.—DR. G. H. WILSON in *Summary*.

**Hopeless Pyorrhea Teeth.** Wherever teeth have lost practically all their solid, bony union, when nothing but a ligamentous attachment is left, I think it best to extract those teeth, because of the impracticability of restoring them. Still, when teeth are in that condition, they can, many times, be so retained with some permanent retaining appliance that they remain comfortable through life. I say comfortable through life, because I have made them comfortable for fifteen or twenty years, when they were in the first place so loose there was nothing save a ligamentous attachment; but it is almost hopeless to endeavor to maintain such teeth in a comfortable condition for any length of time, if the environment is not kept in a sanitary condition. In cases where that is impossible, I now sacrifice teeth which cannot be taken care of in the ordinary treatment, because in those cases, the results are disastrous if the teeth are retained, through the infection of the tissue surrounding the sound teeth in the mouth.—DR. J. D. PATTERSON, in *Western Journal.*

**Chloretone in Post-Operative Nausea and Vomiting.** When applied locally chloretone is an admirable antiseptic, and when administered internally it is a valuable hypnotic and sedative.

In the form of Boro-Chloretone it is used in the treatment of wounds, ulcers, and burns, in which it is highly commended by well-known practitioners in this country and abroad. More recently Chloretone has attracted attention as an aid in the production of general anesthesia. For example, Dr. Henry B. Ingle, anesthetist in the Gynecean Hospital, Philadelphia, administered five grains of Chloretone every fifteen minutes for three doses, or ten grains in one dose one hour before beginning the administration of the general anesthetic. Please note the result: "With such a preliminary medication the patient is less nervous, more easily anesthetized, the amount of narcotic is less, and what is of great importance, the patient "dozes" quietly after recovery from the anesthetic, and post-narcotic nausea and vomiting are greatly decreased."—*Therepeutic Notes.*

**To Mount Carborundum Stones.** Tiny carborundum stones are very useful, and may be successfully mounted, to withstand friction afterwards, without the loosening which always follows the use of shellac varnish.

Carefully fit a worn bur to each stone, laying them in a position to be handled quickly. Puncture several holes, at good distance apart, in a paper box cover, about one inch in depth, to receive the support burs when mounted. Then proceed to mix to a soft consistency a suitable amount of oxyphosphate of copper cement.

Hold stone in left hand, bur in right, work in a small amount of cement to bottom of aperature in stone, add a little more cement on end of bur, push firmly into position—

set in box cover—true up quickly with fingers, and proceed to next stone. Allow cement to harden over night, then place each mounted stone in dental engine, and as it revolves quickly, hold sharp turning tool against any surplus cement, trimming neatly. With practice, one can mount nearly one dozen stones with one mixture of cement.—MARY E. BLAKE, in *Brief*.

**Application of Method to Sensitive Dentine.** For obtunding sensitive dentine, a small quantity of the cocaine solution is taken into cylinder or barrel of syringe, and tapered nozzle placed in drilled aperature. Test to see if there is a perfect joint between tooth and injecting instrument. When contact without leakage has been secured, continued pressure from one to one and one-half minutes will usually be sufficient to send a minute quantity of the obtunding solution through the tubuli onto the surface of the pulp and cause a surface of anesthesia, cutting off sensation from all parts of the tooth corresponding to the surface of pulp covered by cocaine solution. If pressure is continued long enough, the entire pulp will be surrounded by the obtunding solution.

We find that the obtunding effect is only obtained when pulp is reached by obtundent, and it is then so pronounced that applications of extreme heat and cold are not felt as well as the excavation of cavity. For grinding teeth with living pulps for crowning, for those who are guilty of such practice, proceed the same as for excavating sensitive dentine.—DR. C. G. MYERS, in *Amer. Journal*.

**Treating Root Canals.** It may be accepted as a fact that when a root canal has been rendered aseptic by sealing in it such medicants as will destroy the micro-organisms it may have contained that little good, so far as the treatment of the abscess is concerned, is derived from changing dressing after dressing in the canal.

Remedies for the treatment of the pulp, either living or putrescent, should be sealed in the cavity by means of gutta percha, temporary stopping or cement, the latter preferred.

Sandarac varnish and cotton has no place as a filling material or dressing seal in the hands of any clean, careful, competent operator.

The subject of the treatment of the teeth might be continued almost indefinitely, but the suggestions made cover the general principles of the subject, which may be further summed up by saying, be careful, be thorough, be clean.

Apply the principles of general surgery to dental surgery and do not imagine that removing a piece of stinking cotton from a putrescent root canal, and replacing it with another which will soon be as offensive as the first, is a proceeding in any way related to the proper treatment of the teeth.—DR. PLATT, in *Pacific Gazette.*

**Post-Extraction Treatment.**

℞ Orthoform

Europhen ---------------------- aa ℨ j

Liquidi petrolati, q. s. to make a paste. —M.

Sig.—As directed.

You will get much benefit from this, especially country dentists, who have to do a great deal of extracting, in allaying the pain after the extraction; if you have separated the process, or if the process is attached to the cementum of the root; or if you have had to go down and expose the process more or less; in any of those conditions from which you have severe pain you can stop that pain almost like magic by its use. Now, it is claimed by some that orthoform is a disinfectant. It is not a good disinfectant, but is an excellent local anesthetic. You can prolong the anesthetic effect if you add europhen. Europhen is a substitute for iodoform. It is an insoluble product, which, when it comes in contact with water, gradually gives off iodin. Combining these two and adding to that liquid vaseline makes an oily paste. After drying the exposed part apply the oleaginous paste, and you can control the pain. You do not need to be afraid; if the patient lives in the country and cannot come in handily, and you think the pain will not stop by the time the anesthetic effect has passed away, you can give him some of the paste and let it be applied personally. It must be applied to an abraded surface.—J. P. BUCKLEY, in *Dental Review.*

**Stability of Artificial Dentures.** In full uppers and lowers, the stability and usefulness of the appliance depend largely upon placing the artificial teeth over or under the ridge, as the case may be, and in the mouth of a patient anywhere up to forty or forty-five years of age full cusps and normal arrangement of teeth can be resorted to, if the natural teeth approximated the normal; but in the same mouth in old age quite a different articulation should be constructed. The cusps should be ground in some cases quite away in fact, reversing the order by inverting the cusps and changing the sulcate groove, making the grinding surface mill-stone fashion to provide for plenty of lateral motion or have failure of usefulness and comfort as our reward. In malocclusion, such as extensive over or under-bite, not only the presence or absence of cusps plays an important part in the usefulness, but the arrangement of the artificial teeth to each other. The proper relation of the edentulous ridges to each other should first be obtained, then the teeth articulated, so as to be over or under the ridges, even if the finished piece articulates abnormally to a marked degree, is by far better practice than to try to make a normal occlusion out of an abnormal one, and having the appliance operating against all laws of mechanism.—DR. E. M. KETTIG, in *Dentist's Magazine.*

**Arsenical Necrosis.** A young woman had arsenic applied to the pulp of a first molar by a physician, in an attempt to relieve a severe toothache, the arsenical paste being applied and held in place only by cotton. When I saw her two weeks later her face was badly swollen as if from an abscessed tooth, and there was an ugly opening on the neck. On examination I found the second bicuspid, first, second and third molars gone, a fetid odor and the patient unable to open her mouth. Pus was freely discharging from the diseased process, and after washing the mouth I found the gums black in color, but on account of the immobility of the jaw was unable to do much at the first sitting, and as there was already a free opening on the neck, about three inches below the ramus, I instructed her to apply hot applications and return the next day.

The next day she said much pus had been discharged during the night, and was able to open her mouth about half as wide as normal. I scraped away the diseased gum with a sharp bone curette succeeded in removing particles and spiculae of necrosed bone.

This operation was repeated every day for two weeks, not being able to do much each day on account of the severe pain.

At the end of this period the condition was much improved, and seeing that nature was now a ding in throwing off the sequestrum, I dismissed the patient, instructing her to use an antiseptic and healing mouth-wash several times a day, and to return in one week.

On her return I found the swelling nearly gone, very little pus exuding, and did a final curetting, reaching healthy bone. In another month's time the gum had healed beautifully, and there was no remaining sign of the trouble except the scar on the neck.—J. M. BESHOAR, in *Weste n Dental Journal.*

**Amalgam and Cement Filling.** I use soft cement under all amalgam fillings regardless of size and location, and when necessary do not hesitate to cut away sound dentine to provide room for cement. Have followed this procedure for fifteen years, and there is no guess work regarding results. The reasons are: The cement is a non-conductor. It is a wall strengthener. It will absolutely prevent discoloration of tooth if properly used, and by properly used I mean lining the entire cavity except enamel margins with cement. The more cement under any metal filling the better, providing sufficient space is left for a body of metal having required strength. If you have a defective margin, recurrent decay will amount to a trifle when cavity is lined.

Lastly, if cement is properly mixed and used, it will be almost impossible for an amalgam filling to come out. Of course this is not "the feature," as our cavity preparation should be right, but at all times it is not, and if a patient enters my office and announces the fact that one of my amalgam fillings has dropped out, I emphatically deny such assertion, and so far (except one) have been able with my records to prove myself right. In making this statement, I mean where a filling comes out w thin a reasonable time, say five years. Of course, in a longer period, recurrence of decay could destroy entire tooth structure. Possibly fillings may have been lost without my knowledge, as all "failures do not come home to roost."

In crown or buccal cavities, the amalgam is crowded into cement regardless of margins, as their exposure is such that before cavity is filled they can be cleaned if any cement has been crowded out. In proximal cavities where a matrix is used (which should be always) care must be taken of cervical lingual and buccal margins. But, suppose you did crowd cement over margin, it would not be probably any more than that used in an inlay, and we know in such cases the cement stands.—J. P. ROOT, in *Review*.

**Curing Pyorrhea.** I think we are justified in calling a case cured, even though we have not replaced the lost bone or the receded gum, but have merely left a non-irritant surface, a healthy condition of the tissue and the teeth in a reasonably firm condition. There are certain conditions where nature needs arousing. Whereas, in a healthy wound nature will generally throw across her leucocytes and build up walls against infection, the plasma and the other building cells unite that wound by what we call first intention, yet upon a contaminated surface that wound must heal from the bottom; the process of healing is then by granulation, and the tissues must be kept reasonably antiseptic. In one case you may need frequent medication—twice a day or every hour sometimes in an active inflammation, as in the cases of burns. And the like system must be employed in pyorrhea. Many cases of advanced pyorrhea would be benefitted by treatment three times a day, and again many cases, where the cause of the disease has been entirely removed would be utterly ruined if they ever received a second treatment, unless the disease recurred, which would not happen if the place was kept reasonably clean and antiseptic. A little judgment and common sense as to the stage of perfection in the operation and the amount of infection, as to the general care of the mouth and other details, will guide the operator in the future care and necessity of further treatment of the case, and when the necessity of such treatment is determined upon, the patient should be instructed to return at the proper time.

It seems to me that there is a great deal of confusion which need not exist, if we would decide upon preliminaries, as to what we call pyorrhea and as to what we call a cure; if, instead of spending our time and fighting over technical nomenclature, we would confine ourselves to the discussion of the problem of its origin, its treatment, and as to the amount of instruction we should give the patient to prevent its return, we would be better equipped for the successful treatment of these cases —DR. C. L. HUNGERFORD, in *Western Journal.*

**Protection of the Dentist against Specific Infection.** Should the operator inflict even a trivial scratch on himself with an instrument which has become infected by a specific mouth, and which has not been properly disinfected, the result may be highly disastrous. The great advantage of the use of the rubber dam, so far as the patient is concerned, has been already pointed out, but it is when dealing with a mouth which it is known is like'y to prove an active source of infection that the protection to the operator is likewise equally great. •

If, while operating on what is known to be a dangerous mouth, the dentist has the ill-luck to cut his finger with an elevator or other instrument, as has happened before now with the worst results, what can be done? Until a year or so ago, practically nothing.

The published experiments of Metchnikoff and Roux show that it is possible to cause abortion of the chancre following inoculation of syphilitic virus on the eyelid of a chimpanzee, by carrying out mercurial inunction less than one hour after the infecting contact—a curious point being that a solution of sublimate has not the same protective act on. The ointment used in the prophylacitc process is composed of ten parts of calomel to twenty parts of lanolin.

But a more important question remained, viz., whether what was the case with the monkeys would be the case with man. Many persons aware of these researches offered to allow themselves to be inoculated. Dr. Metchnikoff chose a yound medical student, the grandson of an eminent surgeon, who was preparing his thesis on the prophylaxis of syphilis. He had neither hereditary nor acquired taint of the malady. He was inoculated in the presence of Dr. Queyrat of the Cochin, Dr. Saboureau of the Pitie and Dr. Salmon of the Pasteur Institute. Three scarifications with the scalpel were followed by the introduction of the virus. An hour later the inoculated spots were rubbed with the calomel ointment. The same thing was done with a monkey. Neither man nor monkey suffered any evil effects, whereas other monkeys, inoculated at the same time but not treated with the ointment, contracted syphilis. The experimenters discovered, however, that in the case of a monkey the ointment must be applied within twenty hours after inoculation, as otherwise the infection declares itself. Dr. Metchnikoff affirms that if the time limit is respected, immunity is complete. He has since published letters from the three specialists above mentioned, affirming that the young man inoculated showed—more than three months later—no trace of syphilis, and that he had never had it.—(*Dental Surgeon—Dental Cosmos.*

